# A retrospective study of pre-operative fasting times prior to elective or emergency cesarean birth in a large maternity hospital: Lessons to be learned to minimize the fasting time

**DOI:** 10.18332/ejm/188801

**Published:** 2024-07-02

**Authors:** Marja Kaijomaa, Anni Myllymäki, Antti J. Väänänen

**Affiliations:** 1 Department of Obstetrics and Gynaecology, University of Helsinki and Helsinki University Hospital, Helsinki, Finland; 2 Department of Anaesthesiology and Intensive Care, University of Helsinki and Helsinki University Hospital, Helsinki, Finland

**Keywords:** ERAS, enhanced recovery after surgery, cesarean delivery, preoperative fast, oral carbohydrate

## Abstract

**INTRODUCTION:**

When managing elective and emergency cesarean births in the same operating room, unpredictable variations in the start times of the cesareans can prolong fasting periods.

**METHODS:**

The fasting times were retrospectively analyzed on 279 consecutive cesarean births at Helsinki University Women’s Hospital, Finland, during January–February 2023. The fasting times were compared between the urgency groups and for elective cesareans according to their scheduled order on the operation list. The primary outcome was the difference in the fasting times for food and drink, while the secondary outcome was fasting for both food >12 h and fluids >4 h. The fasting times were compared by one-way ANOVA and chi-squared test, respectively. Dichotomous data are presented as unadjusted odds ratios (OR with 95% CI).

**RESULTS:**

Increasing urgency was associated with shorter fasting times. Fasting times for elective cesareans increased with the scheduled order on the daily list. The mean fasting periods (SD) increased from 10.55 h (SD=1.57) to 14.75 h (SD=2.02) from the first to the third cesarean of the day (p<0.01). The unadjusted odds ratio (95% CI) for fasting of the scheduled cesareans to exceed 12 h for solid foods and 4 h for clear fluids was 6.53 (95% CI: 2.67–15.9, p<0.001), for the third and fourth cesareans compared to the first two cesareans of the day.

**CONCLUSIONS:**

When elective and emergency cesareans are performed by the same team, the woman undergoing the third elective surgery of the day should be advised to have breakfast before 5 a.m. at home. While waiting for the operation, a carbohydrate drink should be offered to limit the fast.

## INTRODUCTION

The recent MBRRACE report^1^ on three-year maternal mortality and morbidity, highlighted the importance of managing elective and emergency cesarean cases separately as a key recommendation. This approach streamlines the planning and organization of daily elective cesareans while ensuring improved access to operating rooms for emergency cases.

Aspiration pneumonitis, once a feared complication of obstetric anesthesia, has become rare due to advances in pre-operative fasting practices, the use of non-particulate antacids, and the increased adoption of regional anesthesia techniques for cesarean deliveries^2,3^. However, prolonged fasting times due to delayed surgeries can also have adverse psychological and physiological effects on the mother and even her infant^4-7^. Limiting pre-operative fasting for solid food and fluid is one of the elements of enhanced recovery after surgery (ERAS) guidelines^8^.

According to the guidelines, before elective cesarean deliveries, pregnant women are advised not to eat solid food within 6 hours before the planned surgery time^8^. To avoid the negative effects of fasting, they are advised to drink clear fluids containing carbohydrates up to 2 hours before scheduled surgery. However, most patients with a less urgent emergency cesarean birth are often hospitalized for some time before the decision on operative delivery. While treated in the labor ward during attempted labor or in the antenatal ward due to maternal or fetal treatment, these patients are subject to hospital-provided nutrition or may even be subject to a nil-by-mouth policy before the decision on operative delivery. Thus, these two groups present different challenges regarding their pre-operative fasting^6^.

We hypothesize that treatment of elective and emergency cesarean deliveries in the same operating room causes delays in admission to elective operations. Such delays, especially from the third cesarean onwards, could exceed the recommended pre-operative fasting times by more than two times: exceeding 12 hours for solid food and 4 hours for clear fluids, thus reaching the fasting times associated with a higher incidence of maternal hypoglycemia and ketonuria^5,7^.

To verify our hypothesis, we conducted a study of actual fasting times before cesarean delivery in a busy delivery hospital in Helsinki, Finland, that handled 8208 deliveries in 2022 with a cesarean rate of 24.8%. Cesarean deliveries were performed in two operating rooms and managed by 1.5–2 teams. The primary focus of this study is to determine the impact of urgency and scheduled timing of daily cesarean deliveries on actual maternal fasting times prior to the operation. The secondary objective is to evaluate the incidence of a composite negative outcome consisting of a fasting period that exceeds 12 hours for solid food and over 4 hours for clear fluids in various urgency categories and among pregnant women admitted for planned cesarean delivery at different times of the day.

## METHODS

### Study design and setting

This retrospective cohort study on the actual fasting times for clear fluids and solid food was conducted on parturients coming for elective or emergency cesarean birth in the Helsinki University Women’s Hospital, Helsinki, Finland, between 1 January and 28 February 2023.

Institutional instructions on fasting for solid food and fluids before an elective cesarean delivery are provided to pregnant women in both written and verbal formats. The day before the operation, the pregnant women are informed of their scheduled time of the operation and the advised time of arrival at the hospital. The first two scheduled cesareans are asked to arrive at 7 a.m., while the next two are advised to arrive at 9 a.m. Five cesareans are sometimes scheduled on the same day, but efforts are made to reschedule the fifth cesarean to a less busy day. The operation list for elective cesareans is planned on the preceding week, and daily verifications and evaluations are made due to possible changes in the plans.

According to the guidelines, parturients are advised to avoid solid foods for six hours prior to their scheduled operation time and are encouraged to drink a clear carbohydrate solution (Pre-Op, Nutricia, The Netherlands, 2×200 mL), provided by the hospital during the antenatal visit, up to 2 hours prior to the intended surgery.

All cesarean deliveries, regardless of urgency classification, are performed in two operating theatres and, depending on the availability of operating room staff, are managed by 1.5–2 teams. The scheduled time for elective cesareans is as follows: the first at 8 a.m., the second at 9 a.m., the third at 10 a.m., and the fourth at noon. To ensure immediate availability of the operating theatre and the team for urgent cases in the labor ward, the handling of two simultaneous cesareans is avoided.

### Study population, recruitment, and inclusion criteria

The study population consists of all cesarean births over a two-month period from January to February 2023, including 1270 deliveries, of which 331 were cesarean births. During the study period, the median number of elective cesarean deliveries scheduled during normal office hours (8 a.m. to 4 p.m.) from Monday to Friday was 3 (range: 1–5).

Fasting times were retrospectively collected from pre-operative checklists, with credible documentation available for 279 of the 331 (84%) cesarean deliveries. Most of the missing data involved category I cesarean deliveries, i.e. operations with a maximum delivery target time of 30 minutes. The 279 parturients with sufficient fasting data were included in this study.

### Categorization tools

A modified Lucas classification was utilized for urgency categorization: Cat I (target delivery time in 30 minutes, including immediate deliveries with a target time of fewer than 10 minutes), Cat II (in 60 minutes), Cat III (more than 60 minutes), and Cat IV (elective cesarean delivery)^9^. Any elective operation postponed due to an intervening emergency case or an emergency case waiting for an available operating room was classified as delayed. Longer than expected operation times for previous cesarean deliveries were not considered to cause delays.

For the analysis, the normal working hours period is considered as Monday to Friday between 8 a.m. and 4 p.m., excluding public holidays.

### Ethics

The study plan was approved by the Institutional review board for Helsinki and Uusimaa hospital district (board for Obstetrics and Gynecology) which granted the permission (HUS/730/2022) for the completion of the study on 2 March 2022. Due to the retrospective nature of the study, the requirement for written informed consent was waived based on the Finnish law on medical research (488/1999).

### Statistical analysis

Based on the calculation of the group size, a minimum of 21 and 29 pregnant women per group were required to detect fasting time differences that exceeded one standard deviation (2 hours) and a potential double of the composite secondary outcome rate (solid food fasting for 12 hours and clear fluid fasting for 4 hours), respectively, at alpha=0.05 and beta=0.10.

Statistical analyses were performed using SPSS version 29 (IBM, USA). Continuous variables were compared using one-way ANOVA with Bonferroni post-tests for multiple group comparisons; p<0.05 was considered statistically significant unless adjusted for multiple tests (p<0.0125 for comparisons in four groups). Bivariate data were compared by using the chi-squared test and ordinal data by the Mantel-Haenszel test with p<0.05 considered statistically significant. Odds ratios are presented as unadjusted with a 95 % confidence interval (CI) unless specifically otherwise stated for the logistic regression analysis. For the correlation analysis, the admission and fasting times were calculated as decimal hours from the last oral intake or midnight, respectively, and correlations between the continuous parameters are expressed as unadjusted Pearson correlations.

To determine the effect of delays in the operating room list on the fasting parameters, a logistic regression analysis was performed for the operations done during office hours. The comparisons were made according to urgency category and delayed status. Since the emergency cesarean deliveries in categories I and II were relatively few during office hours and practically not affected by delays due to their time limit for completion, the effect of delays was analyzed on the less urgent category III and elective category IV cesareans, which compete with each other for the same operative resources. The correlations between the admission time and delay status were checked. The binary outcome variables were fasting over 12 hours for solid food, over 4 hours for fluids, or their combination, which represented a 100% increase in the ERAS-recommended fasting times.

## RESULTS

Most elective cesarean deliveries were performed during normal business hours between Monday and Friday and between 8 a.m. and 4 p.m. During these hours, 104 (67 %) of the 156 cesareans performed were elective.

### The effect of the urgency category on fasting times

The urgency of cesarean operations significantly affected the average fasting times, with Cat I emergency operations associated with the shortest fasting periods for both solid food and fluids. The distribution of fasting times by urgency categories is shown in Table 1. The elective cesarean cases showed the longest mean fasting time for solid foods, which exceeded 12 hours, while the longest mean fasting time for clear fluids was observed in the least urgent (Cat III) emergency cases.

**Table 1:** The effect of urgency category on the fasting times, a retrospective analysis of 279 consecutive cesarean births admitted at any time of the day in the Helsinki University Women’s hospital in 2023

	*Urgency category*				*p*
	*I n (%)*	*II n (%)*	*III n (%)*	*IV n (%)*	
**Total**, n	45	46	78	110	
**Fasting** (h)					
Solid food, mean (SD)	8 h 8 min (5 h 1 min)*	12 h 20 min (10 h 56 min)	11 h 56 min (4 h 36 min)	13 h 4 min (3 h 25 min)	<0.01
Clear fluids, mean (SD)	5 h 15 min (4 h 16 min)	6 h 48 min (3 h 59 min)	7 h 48 min (4 h 18 min)*	5h 27 min (3 h 3 min)	<0.01
**Fasting**					
Solid food fast > 12 h	10 (22)	19 (41)	37 (47)	62 (68)	<0.01
Clear fluid fast > 4 h	22 (53)	33 (72)	65 (83)	69 (63)	0.495
Solid food > 12 h and fluid > 4 h	5 (11)	15 (33)	28 (36)	54 (49)	<0.01
**Admission during office hours**	9 (20)	12 (26)	31 (40)	104 (95)	<0.01
**Delayed admission**	0 (0)	6 (13)	23 (30)	54 (49)	<0.01
**Umbilical artery sample**, n	44	46	76	105	
pH, mean (SD)	7.27 (0.09)*	7.31 (0.05)	7.31 (0.05)	7.32 (0.06)	<0.001
BE, mean (SD)	-1.95 (3.36)*	-0.12 (1.97)*	-0.08 (2.34)*	1.18 (2.23)	<0.001

P-values for one way ANOVA for intergroup comparison.

* Bonferroni post-test p<0.0125 against the category IV (elective) cesareans.

Irrespective of the time of admission to the operating room, the unadjusted odds ratio of fasting for 12 hours for solid foods was 2.53 (95% CI: 1.54–4.14, p<0.001), and for 4 hours for clear fluids was 0.65 (95% CI: 0.39–1.08, p=0.096), in elective cases compared to non-elective cases. The similar unadjusted odds ratio for the combined adverse fasting outcome (fasting to exceed 12 hours for solid food and 4 hours for clear fluids) was 2.43 (95% CI: 1.47–4.01, p<0.001) in elective cases compared to emergency cases.

### The effect of admission time to operating room on fasting times

**Figure 1:**
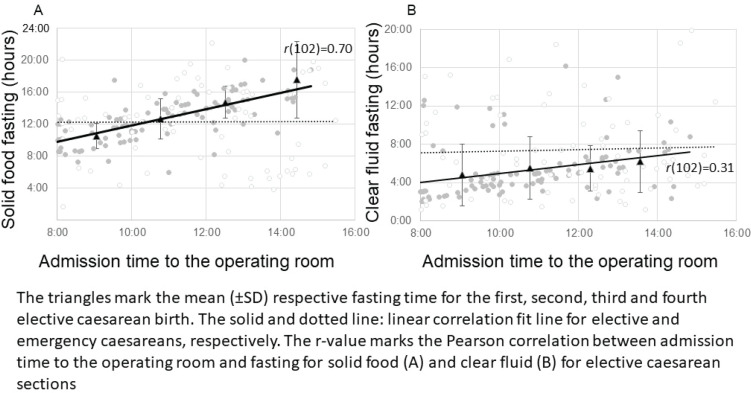
Fasting for solid food and clear fluids in 104 elective (open dots) and 52 emergency (shaded dots) cesarean births in Helsinki University Women’s hospital, January–February 2023

Compared to operations that were carried out outside office hours, the unadjusted odds ratio for fasting time to exceed 12 hours during office hours was 2.48 (95% CI: 1.48–4.15, p<0.001). During office hours, the solid food fasting time for elective cesarean deliveries was positively correlated with the time of admission [Figure 1A; r(102)=0.70, p<0.001], while in emergency cases, there was no correlation [Figure 1A; r(50)= -0.10, p=0.496]. A similar but weaker correlation was observed for fasting for clear fluids, with elective cases showing a positive correlation between admission to the operating room and fasting for clear fluids [Figure 1B; r(102)=0.31, p=0.002], while this was not observed in emergency cases [Figure 1B; r(50)= -0.05, p=0.718].

Following the correlation between fasting status and the time of admission to the operating room, the proportion of women who exceed recommended fasting times by 100% increases according to the planned order on the daily cesarean list for elective cases (Mantel-Haenszel p<0.001, Table 2).

**Table 2:** The effect of order on the daily operating list for category IV cesarean births on the fasting times, a retrospective analysis of 105 consecutive elective caesarean births scheduled during office hours in the Helsinki University Women’s hospital in January–February 2023

	*1st n (%)*	*2nd n (%)*	*3rd n (%)*	*4th n (%)*	*p*
**Total**, n	39	32	25	9	
**Arrival time at operating room** (range)	9:03 (7:58–12:15)	10:46 (9:17–12:59)	12:31 (10:40–17:36)	14:26 (12:36–21:29)	
**Fasting** (h), mean (SD)					
Solid food prior to admission	8 h 29 min (1 h 36 min)	8 h 54 min (2 h 22 min)	11 h 14 min (1 h 44 min)*	12 h 10 min (2 h 42 min)*	<0.001
Solid food prior to operation	10 h 33 min (1 h 34 min)	12 h 41 min (2 h 31 min)*	14 h 45 min (2 h 1 min)*	17 h 36 min (4 h 47 min)*	<0.001
Clear fluids prior to operation	4 h 47 min (3 h 7 min)	5 h 29 min (3 h 7 min)	5 h 30 min (2 h 22 min)	7 h 17 min (3 h 13 min)	0.084
**Fasting prior to operation**					
Solid food fast >12 h	10 (26)	21 (66)	23 (92)	9 (100)	<0.001
Clear fluid fast > 4 h	16 (41)	22 (69)	19 (76)	8 (89)	<0.001
Solid food >12 h and fluid > 4 h	5 (13)	19 (59)	18 (72)	8 (89)	<0.001
**Admission during office hours**	39 (100)	32 (100)	24 (96)	8 (89)	<0.001
**Delayed admission**	15 (39)	17 (53)	13 (52)	5 (56)	0.095
**Umbilical artery sample**					
pH, mean (SD)	7.31 (0.04)	7.32 (0.08)	7.32 (0.05)	7.35 (0.05)	0.325
BE, mean (SD)	1.68 (2.12)	0.92 (2.68)	1.13 (1.40)	-0.48 (2.34)	0.101

P-values for one way ANOVA for intergroup comparison.

* Bonferroni posttest p<0.0125 against the first elective caesarean on the daily list.

### Incidence of delays in operating room admission

According to the operating protocols at our hospital, Cat I emergency cesareans were immediately admitted to the operating room without delay throughout the day. However, for Cat II-IV cesareans, there was an increasing trend in delays, as shown in Table 1 (Mantel-Haenszel p<0.01). During office hours, the incidence of delays in Cat I-II emergency cesareans was 5/21 (24%), while the corresponding incidence of delays was 13/31 (42%) in Cat III emergency cesarean deliveries and 50/105 (48%) in the elective cesarean deliveries scheduled for office hours.

From the clinical point of view, Cat I-II emergency cesareans can be seen as those generating delays that accumulate as the daily operating list proceeds, thus affecting the operations done in the afternoon. The regression analysis for the delays in the Cat III emergency and Cat IV elective cesareans shows that there is a significant positive correlation between the admission time (in hours) after 8 a.m. and the incidence of delays (Tables 3 and 4).

**Table 3:** Regression between admission time (hours from 8 am onwards), the incidence of delays and their effect on fasting outcomes for the 31 Cat III emergency cesareans admitted during office hours at the Helsinki University Women’s hospital in January–February 2023 (N=31)

	*B (SE)*	*p*	*χ^2^*	*df*	*R^2^*
**Logistic regression for the incidence of delays as predicted by the admission time after 8 am**					
Delays (vs time)	0.454 (0.198)	0.022	6.687	1	0.261
**Logistic regression for the fasting over 12 hours for solid food; cofactors: delay status and admission (hours past 8 am) as cofactors**					
Time past 8 am (h)	-0.133 (0.186)	0.475			
Delayed vs Not delayed model	2.231 (1.015)	0.028 0.049	6.012	2	0.239
**Logistic regression for fasting over 4 hours for fluids; cofactors: delay status and admission (hours past 8 am)**					
Time past 8 am (h)	0.223 (0.290)	0.441			
Delayed vs Not delayed model	-0.919 (1.342)	0.493 0.681	0.769	2	0.046
**Logistic regression for fasting over 12 hours for food and over 4 hours for fluids; cofactors: delay status and admission (hours past 8 am)**					
Time past 8 am (h)	-0.032 (0.183)	0.860			
Delayed vs Not delayed model	1.574 (0.881)	0.074 0.135	4.011	2	0.162

SE: standard error. Nagelkerke R^2^ value.

**Table 4:** Regression between admission time (hours from 8 am onwards), the incidence of delays and their effect on fasting outcomes for the 105 elective caesareans planned during office hours at the Helsinki University Women’s hospital in January–February 2023 (N=105)

	*B (SE)*	*p*	χ*^2^*	*df*	*R^2^*
**Logistic regression for the incidence of delays as predicted by the admission time after 8 am**					
Delays (vs time)	0.814 (0.161)	<0.001	41.402	1	0.435
**Logistic regression for the fasting over 12 hours for solid food; cofactors: delay status and admission (hours past 8 am) as cofactors**					
Time past 8 am (h)	0.975 (0.208)	<0.001			
Delayed vs Not delayed model	-1.108 (0.615)	0.072 <0.001	39.101	2	0.420
**Logistic regression for fasting over 4 hours for fluids; cofactors: delay status and admission (hours past 8 am)**					
Time past 8 am (h)	0.479 (0.158)	0.003			
Delayed vs Not delayed model	0.646 (0.531)	0.224 <0.001	24.675	2	0.285
**Logistic regression for fasting over 12 hours for food and over 4 hours for fluids; cofactors: delay status and admission (hours past 8 am)**					
Time past 8 am (h)	0.760 (0.170)	<0.001			
Delayed vs Not delayed model	-0.515 (0.546)	0.345 <0.001	33.710	2	0.366

SE: standard error. Nagelkerke R^2^ value.

### The effect of delays on overall fasting times

Operation delays during office hours were associated with an increase in fasting times for solid food from 12.05 h (SD=2.80) to 13.33 h (SD=2.57) (p=0.017) and for clear fluids from 4.53 h (SD=2.55) to 6.18 h (SD=2.98) (p=0.003). The unadjusted odds ratio of fasting for 12 hours for solid food and 4 hours for clear fluids was 2.62 (95% CI: 1.36–5.03, p=0.004), for delayed cases compared to cases without delay.

Delayed operations could particularly affect less urgent emergency cases (Cat III), which are kept on standby while waiting for their operation time. The combined association of delays and admission time, however, show that while the incidence of delays increases towards the end of working day, neither the admission time nor the delays correlate with the adverse fasting outcomes except for the admission time adjusted higher incidence of fasting for over 12 hours for solid food if the operation is delayed (Table 3).

In contrast to the Cat III emergency operations, in the elective operations, all fasting outcomes: fasting over 4 hours for fluids and over 12 hours for solid food and their combination, are significantly correlated with the admission time to the operating room even when adjusted for the potential delayed operation-status (Table 4). The logistic regression analysis indicates that the delay status itself was not a significant cofactor for adverse fasting outcomes (Table 4).

### The effect of anticipated operation time on prehospital fasting

The first two cesareans of the day had an average fasting time of 8.68 h (SD=1.98) before admission at 7 a.m., while the following two had an average fasting time of 11.48 h (SD=2.03) by their admission time at 9 a.m. (p<0.001). Approximately 10% of the parturients scheduled for the second to fifth cesareans received clear oral fluids after admission. The time of admission to the hospital did not affect the fasting time of fluids, which showed an average fasting of 2.52 h regardless of the time of arrival.

### Impact of maternal fasting on infant acid-base balance

In elective cesarean births, there was a negative correlation between umbilical arterial pH and maternal fasting for clear fluids [r(103)= -0.24, p=0.013]. Infants born after maternal fasting for over 4 hours for clear fluids showed a lower umbilical artery pH (7.31 ± 0.06) compared to those born after maternal fasting for less than 4 hours, pH (7.34 ± 0.05) (p=0.018). However, no similar correlation was observed between pH and fasting for solid food.

## DISCUSSION

Our retrospective study shows that prolonged fasting prior to cesarean birth remains a problem despite international and institutional guidelines, patient education, and the provision of carbohydrate drinks by the hospital during an antenatal visit for the parturients to drink at home prior to arrival at the hospital. Among the identified risk factors for prolonged fasting were: elective cesarean birth, operation during office hours, and not being the first scheduled cesarean of the day. Meanwhile, prolonged fasting for fluids appears to affect the less urgent (Cat III) emergency cesarean births compared to elective cesareans.

Limiting fasting prior to cesarean delivery, particularly in elective cases, is essential to alleviate hunger, anxiety related to the procedure, and post-operative gastric pain^10,11^. Given that cesarean deliveries are usually performed under regional anesthesia without anxiolytic premedication, and it may be the first surgical event for the parturient, any safe intervention to alleviate potential anxiety is beneficial^12^.

The current study highlights that planning elective and emergency cesareans to be performed within the same operating rooms and managed by the same team, results in operational delays as the typical working day progresses. These delays primarily affect elective and less urgent emergency procedures, leading to later admission to the operating theatre and, therefore, to extended fasting periods for both solid food and clear fluids. It is noteworthy that even though delayed operation-status does not appear to associate itself with prolonged fasting, it does, by definition, result in later admission to the operating room, which was highly correlated with the longer fasting times in elective cesarean deliveries and may partially explain the nearly 8-hour fluid fast in the less urgent Cat III cesarean deliveries (Table 1).

Previous studies have shown that consumption of clear carbohydrate drinks within 4 hours prior to cesarean birth can reduce the incidence of ketones in the urine at the time of surgery^5^. In our study population, 69% of all expecting mothers had not consumed anything within 4 hours of their operating room admission. Despite reported challenges related to compliance with carbohydrate drink intake^13^, a considerable proportion (72%) of our elective parturients consumed their provided carbohydrates within two hours before hospital admission.

While carbohydrate loading for elective cesarean cases appears to function relatively well, this study revealed greater challenges in oral fluid consumption for less urgent emergency (Cat III) cesarean deliveries. Multiple factors are likely to affect fluid fasting in these cases: operations are typically performed due to prolonged labor or suspected infections, and patients may be hospitalized for prolonged periods before the decision on operative delivery. These patients may be subject to a ‘nil-by-mouth policy’ and end up with a long fasting period for both food and drink, just in case an operative delivery is needed abruptly or before the actual decision to operate. A high rate (85%) of use of neuraxial analgesia combined with intravenous crystalloid infusion, potentially leads to an oversight in the availability of carbohydrate-containing oral fluids for these pregnant women.

This study reveals several points that should be addressed if pre-operative fasting times are to be limited. To minimize pre-operative fasting times for non-elective cases, it is crucial to provide oral carbohydrate-containing fluids at regular intervals. A can of carbohydrate drink should be provided for women if the waiting time for admission to the operating room is expected to exceed 2 hours, at least if the procedure is delayed by a more urgent operation coming to the theatre. The average operative time from incision to closure in this study was 51 minutes. Considering the time required for the regional anesthesia and transfer of the parturient, it can be estimated that a cesarean case entering the operating room will postpone the next-in-line cesarean by a minimum of 1.5 hours – at least outside office hours.

Although fluid intake can be controlled more precisely on the basis of the operation theatre occupancy, oral intake of solid food cannot be controlled as dynamically. At the time of admission, 93% of the women who arrived at 7 a.m. had already fasted for more than 6 hours. Surprisingly, those arriving at 9 a.m. had fasted for more than 2 hours longer. Improving the information regarding fasting for elective cesarean cases is essential, and elective cesarean cases would likely benefit from more precise information. The key element to limit fasting for solid food is information about oral food intake prior to arrival at the hospital. Pre-operative fasting times in these ranges have been previously reported following a patient education program on pre-operative fasting prior to elective cesarean delivery^13^.

Although admission to the operating room was scheduled at 10 a.m., only 4 of 25 (16%) of the third daily cesarean cases entered the operating room, on average, 9 minutes before 11 a.m. The median entry time was markedly delayed to 12:30 p.m. Thus, it would be advisable for the third cesarean of the day to have a regular breakfast before 5 a.m. Our current operating procedures cannot realistically handle five elective daily cesareans. Attempting such a list will lead to excessive fasting times for pregnant women. Analysis of our data shows that four cesareans were performed in the operating room within business hours in 39 of 41 (95%) cases while trying to perform five cesareans is not realistic. If necessary, the fifth cesarean of the day should have breakfast at home before 8 a.m. or, ideally, the operation should be planned for another day.

Prolonged fasting times have been suggested to affect not only the pregnant woman but possibly also their newborn. Fasting times that exceed 8 hours for food and 2–6 hours for fluids have been associated^4^ with a lower umbilical artery pH, albeit this finding has not been reported in a subsequent study^7^. In our cohort, we observed a negative trend in arterial pH with increasing fasting time for oral fluid intake. However, fasting time for solid food did not show a significant association with changes in umbilical artery pH. The association between fluid intake and umbilical arterial pH is unlikely to be strong, and the overall size of the effect on pH appears clinically insignificant.

Although various factors, such as maternal hemodynamics during surgery and the time from uterine incision to delivery, influence the pH of the umbilical artery of the newborn, the outcome of the newborn should be addressed in future studies on pre-operative fasting.

### Strengths and limitations

Our study has some limitations due to its retrospective nature. The number of parturients in the Cat III (less urgent emergency cesarean category) was relatively small, and therefore, the lack of effect of delays on the fasting outcomes should be considered with some caution. Regarding overall data collection, while the fasting data are gathered from the pre-operative anesthesia checklist system, some pregnant women may have received undocumented fluids. Furthermore, it remains possible that some pregnant women may have consumed food or drink without informing the personnel, given the language barrier between pregnant women and the personnel in many cases. Furthermore, maternal and infant glucose levels are not routinely monitored and, therefore, were unavailable for this study. The routine documentation of the pre-operative fasting in the patient journalling system, the fact that the parturients and the personnel have been educated about the need to limit the fasting time and that the parturients coming for the elective operation receive the pre-operative glucose drinks from the hospital can be considered strengths of our study.

## CONCLUSIONS

Even when both the parturients and the personnel have been informed about limiting the pre-operative fast time and the parturients have consumed their provided fluids prior to arrival to the hospital, the actual pre-operative fasting for both solid food and fluids appears to remain a significant problem that needs to be addressed. The actual pre-operative fasting times should be monitored, and care should be taken to limit the fast to the extent possible. This study reveals three key points affecting the fasting times that should be addressed. First, the actual admission times to the operating room may differ significantly from the planned admission times, particularly if the elective operations are done by the same staff and theatres as emergency cases. To address this, either the elective operations should be done separately from the emergency cases, or the fasting instructions for the elective cases should be based on audited actual operating room admission times. Second, the parturients coming for an elective cesarean delivery should be more explicitly informed to eat real food some hours before their arrival at the hospital, thus keeping the fasting times before the operation closer to 6 hours than 12 hours. Third, while the parturients are waiting for their operation, a clear carbohydrate drink should be offered to them up to 2 hours before the realistically anticipated admission to the operating room.

## Data Availability

The original data presented in support of this work can be requested in anonymized form from the corresponding author following a reasonable and justified request.
